# Deep Sedation and Atrioventricular Nodal Reentry Tachycardia Ablation

**DOI:** 10.5812/cardiovascmed.15032

**Published:** 2013-10-28

**Authors:** Abolfath Alizadeh-Diz

**Affiliations:** 1 Cardiac Electrophysiology Research Center, Rajaie Cardiovascular Medical and Research Center, Iran University of Medical Sciences, Tehran, IR Iran

**Keywords:** AVNRT ablation, Arrhythmias, Cardiac

Cardiac ablation is an invasive procedure requiring conscious and deep sedation for immobility and analgesia. AVNRT ablation generates acute pain during the radiofrequency application. Although the success rate is high, the procedure is uncomfortable for patients who must remain motionless. Sedation in EP lab is needed for several purposes such as providing anaesthesia and airway management for cardio version during EP study and induction of atrial fibrillation, pain management, and haemodynamic monitoring. The best anaesthetic agent should not alter Electrophysiological properties including SA node, AV node functions, conduction velocities, and refractoriness. Most anaesthetic agents have not been studied in the context of EPS and most of our data were based on animal and laboratory experiments. A few investigators reported that anesthetic agents have no significant clinical effects on conduction system (1, 2). There are some reports concerning non-inducibility of AVNRT after intravenous sedation in pediatrics (3). Fazelifar et al. investigated inducibility of AVNRT and electrophysiological properties after deep sedation (4). Anesthesia with benzodiazepines and opiates used in a repeated bolus fashion can easily be provided without influencing accurate EP testing and/or arrhythmia induction. There are no known specific side-effects of benzodiazepines on cardiac conduction (1). Propofol and benzodiazepine may change heart rate via baroreflex regulatory mechanisms (5, 6). The authors demonstrated that AVNRT induction is not related to anesthetic agents and it could be performed safely in all patients who undergo slow pathway induction. Induction and ablation of reentrant arrhythmias such as AVNRT can be performed safely after deep sedation. Ablation after sedation in other supra-ventricular tachy-arrhythmias requires another well-designed randomized trial.
